# Isolation of a Halogen-Bonded Complex Formed between Methane and Chlorine Monofluoride and Characterisation by Rotational Spectroscopy and Ab Initio Calculations

**DOI:** 10.3390/molecules24234257

**Published:** 2019-11-22

**Authors:** Anthony C. Legon, David G. Lister, John H. Holloway, Devendra Mani, Elangannan Arunan

**Affiliations:** 1 School of Chemistry, University of Bristol, Cantock’s Close, Bristol BS8 1TS, UK; 2 Dipartimento di Chimica Industriale, Universita di Messina, Casella Postale 29, 1-98166 San’Agata di Messina, Italy; 3 School of Chemistry, University of Leicester, University Road, Leicester LE1 7RH, UK; jhh2@leicester.ac.uk; 4 Department of Inorganic and Physical Chemistry, Indian Institute of Science, Bangalore 560012, India; devendramani@outlook.com (D.M.); arunan@iisc.ac.in (E.A.)

**Keywords:** halogen bond, microwave spectroscopy, atoms-in-molecules calculations, internal rotation of methane

## Abstract

A halogen-bonded complex formed between methane and chlorine monofluoride has been isolated in the gas phase before the reaction between the components and has been characterised through its rotational spectrum, which is of the symmetric-top type but only exhibits *K* = 0 type transitions at the low effective temperature of the pulsed-jet experiment. Spectroscopic constants for two low-lying states that result from internal rotation of the CH_4_ subunit were detected for each of the two isotopic varieties H_4_C···^35^ClF and H_4_C···^37^ClF and were analysed to show that ClF lies on the symmetry axis with Cl located closer than F to the C atom, at the distance *r*_0_(C···Cl) ≅3.28 Å and with an intermolecular stretching force constant *k_σ_* ≅ 4 N m^−1^. Ab initio calculations at the explicitly correlated level CCSD(T)(F12c)/cc-pVTZ-F12 show that in the equilibrium geometry, the ClF molecule lies along a *C*_3_ axis of CH_4_ and Cl is involved in a halogen bond. The Cl atom points at the nucleophilic region identified on the *C*_3_ axis, opposite the unique C–H bond and somewhere near the C atom and the tetrahedron face centre, with *r*_e_(C···Cl) = 3.191 Å. Atoms-in-molecules (AIM) theory shows a bond critical point between Cl and C, confirming the presence of a halogen bond. The energy that is required to dissociate the complex from the equilibrium conformation into its CH_4_ and ClF components is only *D*_e_ ≅ 5 kJ mol^−1^. A likely path for the internal rotation of the CH_4_ subunit is identified by calculations at the same level of theory, which also provide the variation of the energy of the system as a function of the motion along that path. The barrier to the motion along the path is only ≅20 cm^−1^.

## 1. Introduction

It was established, by a comparison of several series of complexes of the type B···HCl and B···ClF, where B is a Lewis base, that a close parallelism exists among the angular geometries of the hydrogen-bonded and halogen-bonded species for a given Lewis base B [1,2]. This parallelism can be readily rationalised by considering the following set of rules for predicting angular geometries: 

*The equilibrium angular geometry of a hydrogen-bonded complex* B···HX *or a halogen-bonded complex* B···XY *can be predicted by assuming that the internuclear axis of *HX* or *XY* lies either:*

*(1)*  *Along the axis of a non-bonding electron (n) pair carried by the acceptor atom Z of* B *with the order of the atoms* Z···^δ+^H–X^δ-^ *or* Z···^δ+^X–Y^δ-^, *as appropriate, or*
*(2)*  *Alongthe local symmetry axis of a π- or pseudo-π pair if *B* carries no n pairs, with the order of the atoms as* ⁎···^δ+^H–X^δ-^ *or* ⁎···^δ+^X–Y^δ-^, *where ⁎ lies on the π-pair axis or*
*(3)*  *Alongthe axis of a n-pair when* B *carries both n- and π- electron pairs.*

**Figure 1:**
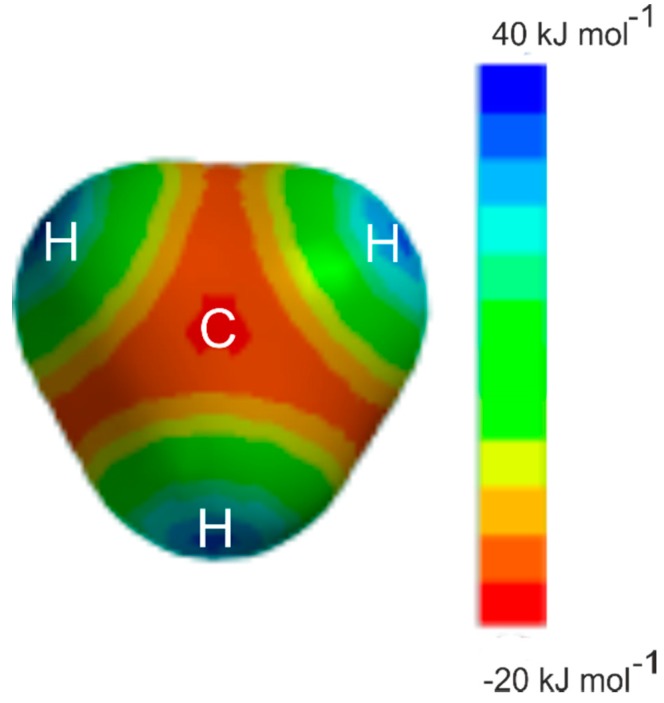
The molecular electrostatic surface potential of CH_4_ at the 0.001 e/bohr^3^ isodensity surface, as calculated at the MP2/6-311++G** level of theory. The view is along a *C*_3_ axis of the molecule. The deepest red region along this axis is at the face centre (almost exactly beneath the C symbol), indicating a nucleophilic (negative, −20 kJ mol^−1^) region of electrostatic potential energy. There are clearly paths to the other three equivalent regions that are only slightly less nucleophilic (orange, −15 kJ mol^−1^) than the face centre region. The blue areas indicate electrophilic regions known as sigma holes (positive +40 kJ mol^−1^).

Examples for which the angular geometries are predicted by Rule 2 include several prototype hydrocarbons, for example, ethyne [3,4], ethene [5,6], allene [7,8], cyclopropane [9,10], and benzene [11,12]. The prototype alkane methane has no occupied π-pair orbitals or n-pair orbitals and hence, appears not to fall within the scope of the rules. Nevertheless, it was shown to form very weak complexes of the symmetric-top type with proton donors HX when X is either CN [13,14] or Cl [15,16] or Br [17], that is with the ^δ+^H atom of HX forming a hydrogen bond to a face centre of the methane molecule with a very low potential energy barrier to the internal rotation of the CH_4_ subunit. Hydrogen fluoride was initially thought to form a complex with methane having a different type of geometry [15], but the rotational spectrum that led to this conclusion arose from H_4_C···H_2_O [18] generated from water in the tank in which the gases were mixed. H_4_C···HF has since been identified [19] through its rotational spectrum and shown to be isomorphous with the other H_4_C···HX. Evidently, the face centre of methane is a nucleophilic region, as may be seen by the molecular electrostatic surface potential (MESP) of methane shown in Figure 1. The MESP was calculated at the 0.001 e/bohr^3^ isosurface at the MP2/6-311++G** level of theory with the Spartan program [20]. The MESP calculated at the MP2(Full)/aug-cc-pVTZ level is given in the Supplementary Material and the results are very similar. It was shown some years ago [21] that HCN forms a hydrogen bond to the centre of the face of ethane to give a complex of *C*_3v_ symmetry. The region near to C, along a *C*_3_ axis and opposite a C–H bond in methane or a C–C bond in ethane, is an example of a local maximum in electrostatic potential which is the site for potential electrophilic attack, which could accept a hydrogen bond [22]. On the other hand, the region just outside each H and along a C–H bond direction in methane is clearly electrophilic (positive, blue) and is an example of what is called a σ hole [23]. Raghavendra and Arunan reported atoms-in-molecules (AIM) theory [24] results on H_4_C···HX (X = F/Cl/OH/SH) complexes and showed that bond critical points were observed for both geometries, showing that CH_4_ can be both a hydrogen bond donor and acceptor [25].

Given the recent growth of interest in the halogen bond and the parallelism of B···HCl and B···ClF angular geometries already referred to, it is of interest to investigate whether methane forms a halogen-bonded complex with ClF that is isomorphous with H_4_C···HCl. In this article, we report such an investigation conducted by rotational spectroscopy and ab initio calculations. Computational results on the isomer CH_4_···FCl geometry, having a C-H···F hydrogen bond, have been included for comparison.

## 2. Experimental and Theoretical Methods

The rotational spectrum of a complex of methane with chlorine monofluoride was observed with a pulsed-jet, Fourier-transform microwave spectrometer of the Balle-Flygare type [26,27], but was modified to include a fast-mixing nozzle [28] to generate the complexes, thereby avoiding a reaction between methane and ClF. A mixture composed of approximately 1% to 2% ClF in argon and held at a stagnation pressure of 3 bar was pulsed via a Series 9 solenoid valve (Parker-Hannifin Corp.Cleveland, ohio, USA.) down the outer of the two concentric, nearly coterminal tubes that form the mixing nozzle. The gas pulse emerging from this nozzle into the evacuated Fabry-Perot cavity of the spectrometer encountered methane that flowed continuously through the inner glass tube of the mixing nozzle into the cavity and complexes H_4_C···ClF were formed at the cylindrical interface of the two gas flows. The methane flow rate was adjusted to give a nominal background pressure of approximately 4 × 10^−4^ mbar at room temperature. When the complexes were between the Fabry-Perot mirrors, they were rotationally polarized by suitably timed pulses of microwave radiation of 1.1 μs duration. The subsequent free-induction decay at rotational transition frequencies was collected, Fourier-transformed, and processed in the usual way. Individual nuclear quadrupole hyperfine components (full-width at a half-height of approximately 15–20 kHz) in the rotational spectrum of H_4_C···ClF arising from the presence of Cl nuclei were well resolved and could be measured with an estimated accuracy of 2 kHz. Rotational transitions of both isotopologues H_4_C···^35^ClF and H_4_C···^37^ClF were readily observed in natural abundance. Unfortunately, flowing the methane through the outer component of the nozzle continuously consumed too much sample to allow expensive isotopically substituted methane samples to be used. The ClF was prepared and purified by the method of Shack and Wilson [29] while natural gas was used, without purification, as the source of methane.

Ab initio geometry optimisations for H_4_C···ClF, H_4_C···HCl and CH_4_···FCl were carried out at the CCSD(T)(F12c) explicitly correlated level of theory [30] with appropriately optimised cc-pVTZ-F12 basis functions [31] by using the MOLPRO program [32]. The potential energy as a function of relative angular orientations of the two subunits (see later) was calculated by optimising all other geometrical parameters at a fixed orientation and repeating this procedure in suitable increments of the angle. The AIM analysis was performed using AIMALL software [33]. The wavefunction that was appropriate to the optimised geometry at the MP2/aug-cc-pVTZ level using the GAUSSIAN electronic structure package [34] was employed in the AIM analysis. Molecular electrostatic surface potentials (MESPs) were calculated at the 0.001 bohr/Å^3^ iso-surface by employing the MP2/6-311++G** level of theory in the SPARTAN electronic structure program [20] and at the MP2/aug-cc-pVTZ level using the GAUSSIAN electronic structure package [34] (see Supplementary Information).

## 3. Results

### 3.1. Determination of Spectroscopic Constants

The observed rotational spectra of the two isotopologues H_4_C···^35^ClF and H_4_C···^37^ClF had the form expected for a symmetric-top molecule in its vibrational ground state, with the quantum number *K* = 0 and a single Cl nucleus on the symmetric-top axis. Two *J* +1 ← *J*, *K* = 0 ← 0 rotational transitions, with *J* = 1 and 2, were identified for each isotopologue within the frequency range (6–20 GHz) of the spectrometer. Each transition exhibited the characteristic quadrupole hyperfine structure arising from a Cl nucleus (spin quantum number *I* = ^3^/_2_) located on the top axis. In fact, for each *J* +1 ← *J*, *K* = 0 ← 0 transition, two almost identical hyperfine patterns were detected close together in frequency compared with the spacing between the two consecutive *J* transitions that were investigated. The two groups of hyperfine transitions of a given *J* were assigned to states with the symmetry labels *A* and *F*, where the nomenclature in use is that introduced by Ohshima and Endo [16] in their analysis of the similar spectrum of H_4_C···HCl. These authors treated the coupling of the angular momentum arising from the internal rotation of the CH_4_ subunit with that of the rotation of the whole molecular framework. They thereby produced an energy level diagram which revealed the correlation between the free-rotor states *j* = 0, 1, and 2 of CH_4_ and various states of the complex as a function of the barrier hindering the free rotation. For H_4_C···HCl, the population at the end of the supersonic expansion appears to be limited to the three lowest internal-rotation states. These states were predicted to belong to symmetry species, *A*, *E*, and *F*, and were shown to have relative intensities of 9:2:5, respectively, when nuclear spin statistical effects were included. The *A* and *F* state transitions correspond to the *K* = 0 components while the *E* state corresponds to *K* = 1 for H_4_C···HCl. The *E* state transitions were readily distinguished by their characteristic *K* = 1 type of Cl-nuclear quadrupole hyperfine structure.

In the case of H_4_C···ClF, a similar behaviour might be expected. The two sets of transitions that were observed for each *J* for H_4_C···ClF are of the *K* = 0 type, as indicated by the nuclear quadrupole hyperfine structure of the *J* = 2 ← 1 and *J* = 3 ← 2 transitions, hence the labels *A* and *F* that are assigned. Presumably, the different nozzle type used here, with different conditions compared with those used for H_4_C···HCl in references 15 and 16, led to a lower effective temperature of the expansion and this coupled with the smaller statistical weight of the *E* state and a somewhat weaker spectrum precluded the observation of the *E* (*K* = 1) state group of transitions. The observed frequencies of the hyperfine components in the *J* = 2 ← 1 and 3 ← 2 transitions for the ^35^Cl and ^37^Cl isotopologues are available in Table S1 as Supplementary Material. The residuals from the final cycles of the fits described below are also given in Table S1. 

Observed frequencies of nuclear quadrupole components of the *J* = 2 ← 1 and 3 ← 2 transitions were fitted in an iterative, least-squares analysis using the Pickett’s SPFIT program [35]. The Hamiltonian that was chosen had the form

*H* = *H*_R_ − ^1^/_6_**Q**(Cl):∇**E**(Cl) + ***I***_F_**M**(F)***J*** (1)

in which *H*_R_ is the familiar Hamiltonian for a semi-rigid, symmetric-rotor molecule in its vibrational ground state. The second term is the energy operator for the interaction of the Cl nuclear electric quadrupole moment **Q**(Cl) with the electric field gradient ∇**E**(Cl) at the Cl nucleus. The third term of Equation (1) accounts for the coupling of the F nuclear spin angular momentum ***I***_F_ to ***J*** by means of the magnetic spin-rotation mechanism, where **M**(F) is the fluorine spin-rotation coupling tensor. The contributions of Cl spin-rotation coupling to transition frequencies can be ignored because the magnetic moment of Cl is 3.2 times smaller than that of F and even the F spin-rotation effect is only just detectable. The four protons of CH_4_ have magnetic moments of a similar magnitude to that of F and lead to a very complicated, largely unresolved hyperfine structure which manifests itself as line broadening and shape distortion on some of the Cl nuclear quadrupole components. 

The matrix of *H* was constructed in the ***I***_Cl_ + ***J*** = ***F***_1_; ***F***_1_ + ***I***_F_ = ***F*** coupled basis and diagonalized in blocks of *F*. For a vibrational ground-state, symmetric-top molecule in a *K* = 0 state, only the components *χ_aa_*(Cl) = *eQ*(Cl) ∂^2^*V*(Cl)/∂*a^2^* and *M_bb_*(F) = *M_cc_*(F) of the two coupling tensors are determinable. Only the quartic centrifugal distortion constant *D_J_* was necessary to produce a fit with an R.M.S. error σ similar to the estimated error (2 kHz) of the frequency measurement. The observable spectroscopic constants that were determined for the two investigated isotopologues of H_4_C···ClF are given in Table 1, along with their standard errors, which were obtained using Kisiel’s PIFORM program [36]. Residuals of the fits are in Table S1.

### 3.2. The Equilibrium Geometry of H_4_C···ClF and Variation of the Potential Energy Function with Internal Rotation of the CH_4_ Subunit

**Figure 2:**
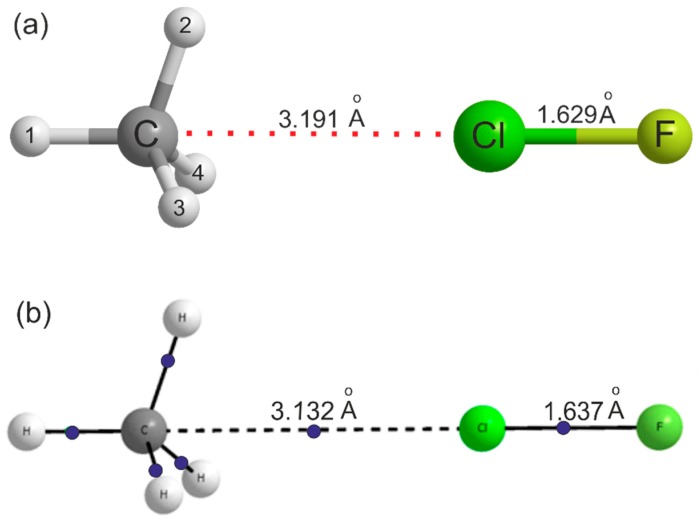
(**a**) The symmetry and geometry of the H_4_C···ClF complex as optimised ab initio at the CCSD(T)(F12c)/cc-pVTZ-F12 level of theory. Further bond lengths and angles are in Table 2. (**b**) The result of an atoms-in-molecules (AIM) calculation conducted on the geometry optimised at the MP2/Aug-cc-pVTZ level using the GAUSSIAN electronic structure package. Bond critical points are indicated by blue dots. The important distances in the complexes calculated at the two levels are shown in the structure and indicate that the results are similar. Note that there is a bond critical point between the CH_4_ face centre and the Cl atom.

For the reason already given, no isotopic substitution in the methane subunit was possible in this study. The main question of interest in H_4_C···ClF is whether the ClF molecule forms a halogen bond to the centre of a face of the methane molecule. ^13^C substitution is unhelpful in that respect and although a single D substitution in the methane subunit in the case of H_4_C···HCl [15] gave a hint of a low barrier to internal rotation of CH_4_, no firm conclusion was possible. Ab initio calculations at the CCSD(T)(F12c)/cc-pVTZ-F12 level of theory reported here reveal that the equilibrium geometry of H_4_C···ClF is as shown in Figure 2a, that is with Cl forming a halogen bond to the centre of a face of the methane molecule. 

Full details of the geometries of H_4_C···ClF, CH_4_ and ClF optimised at this level of theory are given in Table 2. Note that for H_4_C···ClF, there are two optimised geometries listed in Table 2. In column A, the angle ∠H_1_–C···Cl was fixed at 180.00°, but in column B, the angle was released and optimised at 180.39° (see Supplementary Material for the MOLPRO output files). The deviation from 180° in column B results from use of an incomplete basis set. The following discussion refers to the results in column A. The energy difference between the two geometries is 5.0 kJ mol^−1^, which, as expected, is very small. The changes in the methane geometry on complex formations are extremely small, with increases of only 0.001 Å in the length of the three equivalent C–H bonds nearest to the Cl atom and 0.2° in the three equivalent HCH angles. The Cl–F bond lengthens by 0.0036 Å. The equilibrium dissociation energy calculated at the CCSD(T)(F12c)/cc-pVTZ-F12 level is *D*_e_ = 5.5 kJ mol^-1^ and at the MP2/aug-cc-pVTZ level is *D*_e_ = 5.4 kJ mol^−1^, after BSSE correction, thereby confirming that the complex is very weakly bound. The latter value reduces to *D*_0_ = 4.4 kJ mol^−1^ after zero-point energy corrections. For comparison, the corresponding *D*_e_ value and ClF bond lengthening for the van der Waals molecule Ar···ClF are 3.0 kJ mol^−1^ and 0.001 Å, respectively, when calculated at the CCSD(T)(F12c)/cc-pVTZ-F12 level. Figure 2b shows the result of the AIM calculation at the MP2(Full)/Aug-cc-pVTZ level, with bond critical points (bcp) indicated by blue dots. Although the AIM calculations were done at different levels, comparison of the dissociation energy and geometry at these two levels indicate that the major conclusions presented here would not change with the level of calculations. There is a bcp between the C atom in methane through the face centre and the Cl atom in ClF. The electron density *ρ* and the Laplacian of the electron density $\nabla^{2}\rho$ at this bcp are 0.0085 au and +0.0411 au, respectively, thereby confirming the presence of the halogen bond. These may be compared to the values 0.0107 au and +0.0407 au, respectively, for H_4_C···HCl [24]. All the AIM parameters for H_4_C…ClF and H_4_C…HCl complexes are given in Table S2 in the Supplementary Material. For comparison, results for the CH_4_…FCl geometry having a C-H…F hydrogen bond are included as well. The electron density at the bcp for this geometry is slightly smaller than that for the halogen-bonded geometry, 0.0071 au, indicating that the hydrogen bonded complex is weaker than the halogen-bonded geometry that was observed in the experiments. The BSSE corrected interaction energy for this structure is −0.9 kJ mol^−1^ and this structure is not stable when zero-point vibrational energy corrections are taken into account (BSSE+ZP corrected energy is +1.0 kJ mol^−1^). All AIM parameters indicate that the interactions in all these complexes are closed shell in nature and are comparable to other halogen/hydrogen bonded complexes [24]. 

**Figure 3:**
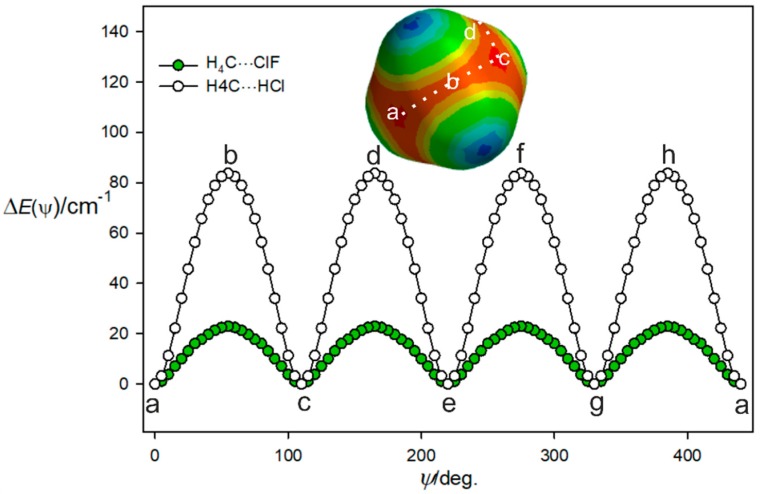
Variation of the energy of H_4_C···ClF with the angular orientation *ψ* of the two subunits that follows the lowest energy path a, b, c, etc. between the four equivalent minima. See the text for a definition of the angle *ψ*.

Given the weakness of the interaction of the ClF and CH_4_ molecules and the fact that methane has four equivalent face centres, it is likely that there is a low barrier to internal rotation of the CH_4_ subunit between the four equivalent equilibrium positions. This can be illustrated by the potential energy as a function of the path shown as a dotted white line on the MESP diagram of methane in Figure 3 and described by an angle ψ. The angle ψ is 0° when the ClF molecules lies along the *C*_3_ axis at position (a) in Figure 3 and the Cl atom is involved in a halogen bond to that face centre. The angle ψ was then increased in 5° steps by rotating the CH_4_ subunit, with the C, Cl, and F nuclei constrained to be collinear at each step and to lie in the plane that contains the initial *C*_3_ axis at position (a), a *C*_2_ axis at position (b), and the next *C*_3_ axis at position (c). The geometry and energy *E*(ψ) were calculated at each step by optimisation of all parameters other than ψ. This path is, thus, along the centre of the red nucleophilic region in the MESP of methane, which is included in Figure 3. The angle ψ = 54.74° corresponds to ClF lying along a *C*_2_ axis of the methane subunit at position (b), where Cl is looking at the tetrahedron edge centre and lies on the *C*_2_ axis. When the ClF molecule is in position (c) and therefore, lies along the next *C*_3_ axis, the angle ψ is 109.47°. The CH_4_ subunit and path then turn, as indicated, to follow the next (identical) nucleophilic routes (c), (d), (e), and so on until all four most nucleophilic centres are sampled. There is clearly a very low potential energy barrier at the centre of each tetrahedron edge along the path. In the complex, the internal motion of the CH_4_ subunit is likely to be constrained mainly to describe the path (a), (b), (c), (d), (e), etc., illustrated in Figure 3 because it is the most nucleophilic route.

**Figure 4:**
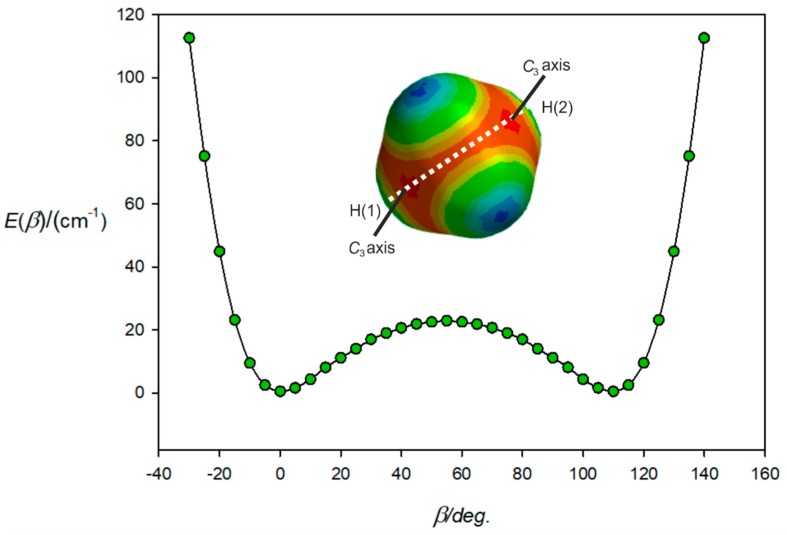
Variation of the energy of H_4_C···ClF with the angle *β* made by the ClF subunit with the C atom of CH_4_ and when ClF lies in the H(1)-C-H(2) plane. At *β* = 0°, ClF lies along a *C*_3_ axis and at *β* = 54.7°, lies along the *C*_2_ axis associated with the edge between atoms H(3) and H(4). At *β* = 109.47°, ClF lies along the other *C*_3_ axis indicated. As *β* further increases, the energy rises until the H(2) atom is encountered.

Figure 4 shows the result of the calculations (with the same constraints of collinearity, but this time defining the angle as *β*) when the path is confined to the H(1)-(a)-(c)-H(2) plane and starts near to one (electrophilic, blue) hydrogen atom H(1), passes through one face centre, through a tetrahedron edge, through another face centre, and finishes near H(2). Clearly it is the high electrophilicity (and therefore, potential energy) near the H atoms that acts to confine the path indicated in Figure 3.

The same procedure as used to produce the potential function for H_4_C···ClF in Figure 3 was followed for H_4_C···HCl, with the calculations again conducted at the CCSD(T)(F12c)/cc-pVTZ-F12 level. The result is included in Figure 3, which shows that the barrier to the motion described is about four times larger than that established for H_4_C···ClF. Presumably, the smaller size of the H atom in HCl means that it can more closely approach each nucleophilic region that is near to the face centre of methane and opposite a C–H bond. Moreover, at point b, the negative region around the Cl atom could have attractive interaction with the positive potential around the H atoms, stabilizing this orientation, also leading to a reduction in the barrier. This is confirmed by NBO calculations, which show the overlap between the lone pairs of electrons in the Cl atom with the C-H anti-bonding orbitals in CH_4_, leading to more stabilization compared to that of the HCl complex. These results are presented in the Supplementary Material.

Figure 4 in the paper by Ohshima and Endo [16] shows the correlation between the energy of the free rotor states *j* = 0, 1, and 2 of CH_4_ and the energies of various states of the complex H_4_C···HCl as a function of the barrier hindering the free rotation of the CH_4_ subunit. Examination of this figure suggests that the lower barrier that exists for H_4_C···ClF is likely to lead to higher energies for the *K* = 1, *E*, and *F* states that correlate with the *j* = 1 and 2 free-rotor states of methane, respectively, than exist for H_4_C···HCl. This is probably another reason why a careful search failed to reveal rotational transitions in *K* = 1 states for H_4_C···ClF. 

### 3.3. Interpretation of the Ground-State Spectroscopic Constant of H_4_C···ClF

Only zero-point spectroscopic constants were obtained from the rotational spectrum of H_4_C···ClF (we use the nomenclature “zero-point” and the subscript 0 for both the *A* and *F* states for convenience in this section because although the *A* state has the lower energy, both states must lie close together). These spectroscopic constants can be interpreted to provide a *r*_0_-type value of the C to Cl distance for each state and another measure of the binding strength, namely the intermolecular stretching force constant *k*_σ_, that is appropriate to the two lowest energy states of this complex. The fact that the difference in *B*_0_ of H_4_C···^35^ClF and H_4_C···^37^ClF is only 1.5 MHz shows that Cl lies close to the centre of mass of the complex and is the atom involved in the (halogen-bond) interaction.

**Figure 5:**
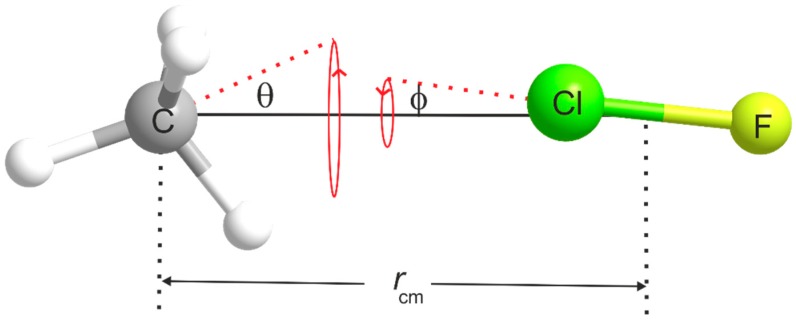
Definition of the distance *r*_cm_ and the angles *θ* and ϕ used to determine the geometry of H_4_C···ClF from the spectroscopic constants recorded in Table 1 and Table 4.

The most significant contributions to the difference between the equilibrium and zero-point moments of inertia of a weakly bound complex such as H_4_C···ClF originate in the effect of the intermolecular bending and stretching modes. The model [37] of the complex commonly used to allow for the contribution of the intermolecular bending modes is shown in Figure 5. The subunits CH_4_ and ClF are assumed to undergo the indicated angular oscillations about their mass centres, with amplitudes *θ* and ϕ respectively. Each oscillation is assumed to be two-dimensionally isotropic and the distance *r*_cm_ between the mass centres is assumed to be fixed. This model, therefore, does not allow for intermolecular stretching. In Figure 5, *θ* and ϕ are the angles made by the *C*_3_ axis of CH_4_ and internuclear axis of ClF, respectively, with the line *r*_cm_. The model relates $I_{b}^{0}$ of the complex to the ground-state moments of inertia $I_{b}^{{\mathrm{CH}}_{4}}$ and $I_{b}^{\mathrm{ClF}}$ of the two components according to

(2) $$I_{b}^{0}\approx \mu r_{\mathrm{cm}}^{2}+I_{b}^{{\mathrm{CH}}_{4}}+\frac{1}{2}I_{b}^{\mathrm{ClF}}\langle 1+\cos^{2}\phi \rangle$$

The $I_{b}^{{\mathrm{CH}}_{4}}$ term is necessarily independent of the angle *θ* because of the *T*_d_ symmetry of methane. A value of 〈$\cos^{2}\phi$〉 can be estimated from the ^35^Cl nuclear quadrupole coupling constant $\chi_{aa}(\mathrm{Cl})$ because the observed value of this quantity is the projection of the free ClF molecule coupling constant $\chi_{0}(\mathrm{Cl})$ [38] onto the *a* axis, averaged over the zero-point motion, i.e., $\chi_{aa}(\mathrm{Cl})=\frac{1}{2}\chi_{0}(\mathrm{Cl})\langle 3\cos^{2}\phi -1\rangle$. This is strictly correct only if the electric field gradient (efg) at the Cl nucleus is unchanged by the presence of the CH_4_ electric charge distribution nearby. The values $\phi_{\mathrm{av}}=\cos^{-1}{\langle \cos^{2}\phi \rangle }^{\frac{1}{2}}$ for H_4_C···^35^ClF and H_4_C···^37^ClF that result are given in Table 3, while the required values of $\chi_{0}(\mathrm{Cl})$ [38] are in Table 4. The $\phi_{\mathrm{av}}$ values are likely to be good estimates given that the octupole moment is the first non-zero moment of the methane electric charge distribution. Thus, $\chi_{aa}(\mathrm{Cl})$ will not be significantly changed by any modification of the efg at Cl due to the presence of methane. When $\phi_{\mathrm{av}}$ is used in Equation (2) together with the appropriate zero-point moments of inertia calculated from the free molecule ground-state rotational constants of CH_4_ [39] and ClF [40] given in Table 4, the distance *r*_cm_ between the centres of mass of CH_4_ and ClF and the distance *r*(C···Cl) = *r*_cm_ − *r* can be calculated, where *r* is the distance from Cl to the ClF mass centre. The results of this procedure are collected in Table 3 for the *A* and *F* states of both H_4_C···^35^ClF and H_4_C···^37^ClF. We note that although *r*(C···Cl) is isotopically invariant within each state, it is considerably longer in the *F* state than in the *A* state.

The intermolecular quadratic stretching force constant *k_σ_* provides a further measure of the strength of the interaction of the subunits CH_4_ and ClF. It is the restoring force for unit infinitesimal displacement along the weak bond direction (the *C*_3_ axis of CH_4_ in this case). When terms higher than quadratic in the potential energy function are neglected and when the components are assumed rigid and unperturbed by complex formation, Millen [41] showed that *k*_σ_ is related to the centrifugal distortion constant *D_J_* of an axially symmetric complex such as H_4_C···ClF by

(3) $$k_{\sigma }=\left(16\pi^{2}\mu B^{3}/D_{J}\right)\left[1-\left(B/B^{{\mathrm{CH}}_{4}}\right)-\left(B/B^{\mathrm{ClF}}\right)\right]$$

where $\mu =\;m_{{\mathrm{CH}}_{4}}m_{\mathrm{ClF}}/\left(m_{{\mathrm{CH}}_{4}}+\;m_{\mathrm{ClF}}\right)$ is a reduced mass. The rotational constants *B, B*^CH4^, and *B*^ClF^ in Equation (3) should be equilibrium values for the complex, CH_4_ and ClF, respectively, as should the centrifugal distortion constant *D_J_*. In the absence of the equilibrium quantities, the corresponding zero-point values from Table 1 and Table 4 were used. The results from Equation (3) are included in Table 4 and we note that within the experimental error generated from the observed *D_J_* values, the *k_σ_* are invariant when ^35^Cl is substituted by ^37^Cl within a given state. However, there is clearly a significant change between the *A* and *F* states, implying that the *F* state is more strongly bound than the *A* state. This observation appears to be in conflict with a distance *r*(C···Cl) that is longer by 0.02 Å in the *F* state (see Table 3), but this conflict probably arises from vibration-rotation coupling resulting from the low barrier to internal rotation of the CH_4_ subunit (see Section 3.2). Nevertheless, the values of *k_σ_* are very small, a result that is consistent with the small value of the dissociation *D*_e_ obtained from ab initio calculations.

## 4. Conclusions

Ab initio calculations at the CCSD(T)(F12c)/cc-pVTZ-F12 level of theory have shown that methane and chlorine monofluoride form a weak complex in which the ClF molecule lies along a *C*_3_ axis of methane, with the electrophilic Cl atom interacting with the most nucleophilic region of methane, namely a region opposite a C–H bond, along a *C*_3_ axis and somewhere between C and a face centre of the tetrahedron. The four identical energy-minimum positions are linked by a relatively low energy path, as indicated by the molecular electrostatic surface potential calculated at the MP2/6-311++G** level, which is in close agreement with the result of calculations at the MP2/aug-cc-pVTZ level (available in the Supplementary Material). The AIM calculations at the latter level show that there is a bond critical point between C and Cl, confirming the presence of the halogen bond. Calculations of the energy of the complex along the low-energy paths between the minima indicate that the barrier presented to the internal rotation of the CH_4_ subunit between the four equivalent minima is only about 20 cm^−1^., which is four times smaller than that similarly calculated for the isomorphous hydrogen-bonded complex H_4_C···HCl. The equilibrium dissociation energy *D*_e_ of the complex is only ≅5 kJ mol^−1^. There is clearly a halogen bond linking the two molecules in this complex, albeit a very weak one.

The rotational spectra of the two isotopologues H_4_C···^35^ClF and H_4_C···^37^ClF that were observed in a pulsed jet at temperatures of approximately 2 or 3 K by using a fast-mixing nozzle (to arrest any reaction between the two components) confirm that the prototype alkane molecule methane does indeed form complexes with chlorine monofluoride in which the Cl atom is closer to the C atom than is the F atom. Moreover, the fact that the rotational spectrum can be detected in two low-lying energy states (labelled *A* and *F*) that almost certainly arise from the internal rotation of CH_4_ between the four equivalent minima is consistent with the potential energy function calculated ab initio. As expected, the *r*_0_-type bond length determined from the rotational constants of the *A* and *F* states are longer than the equilibrium quantity calculated ab initio. Small experimental values of the intermolecular stretching force constant are consistent with the small ab initio values of *D*_e_.

Complexes in which an HX molecule forms a hydrogen bond to a face centre of methane were identified as long ago as the early 1990s for X = HCN [13,14], HCl [15,16], and HBr [17]. The work by Ohshima and Endo [16] concerning the internal rotation of the CH_4_ subunit in H_4_C···HCl was important in establishing this conclusion, as was the investigation of C_2_H_6_···HCN [21], which demonstrated that HCN forms a hydrogen bond to the centre of the face of a CH_3_ group in a situation where a facile internal rotation of ethane between face centres was unlikely. Atoms-in-Molecules theory [24] applied to H_4_C···HX (X = F/Cl/OH/SH) complexes by Raghavendra and Arunan [25] showed that bond critical points exist between H of HX and C of CH_4_. AIM calculations reported here also reveal a similarly placed bond critical point in the isomorphous complex H_4_C···ClF. A question of general interest concerns nomenclature in the context of recent definitions of the hydrogen bond [42] and the halogen bond [43]. The nucleophilic region somewhere between C and a face centre of methane can interact with the electrophilic region near H or Cl in forming H_4_C···HCl or H_4_C··· ClF, respectively [23]. In reference [25], the H_4_C···HX (X = F/Cl/OH/SH) were referred to as stabilised by hydrogen bonds to carbon. The geometries of all H_4_C···HX and H_4_C···ClF that have been investigated so far show that three of the H atoms of CH_4_ are closer to H in HX or to the Cl in ClF than to C and therefore, that such structures could possibly be classified as trifurcated hydrogen bonds instead of hydrogen or halogen bonds if one did not look at the electron density. Recently, however, Frontera and co-workers [44] analysed CH_3_···O contacts in proteins and concluded that these were stabilized by carbon bonds [45] to O and are not trifurcated H bonds.

## Figures and Tables

**Table 1:** Determined spectroscopic constants of H_4_C···^35^ClF and H_4_C···^37^ClF.

Spectroscopic Constant	H_4_C···^35^ClF		H_4_C···^37^ClF	
	State *A*	State *F*	State *A*	State *F*
*B*_0_/MHz	2307.2111(9) ^a^	2287.9319(9)	2305.6624(6)	2286.3818(10)
*D_J_*/kHz	8.83(6)	7.16(6)	8.88(4)	7.16(7)
χ*_aa_*(Cl)/MHz	−142.483(11)	−142.425(8)	−112.322(6)	−112.287(9)
*M_bb_*(F) = *M_cc_*(F)/kHz	2.6(10)	2.9(9)	2.5(7)	2.3(11)

^a^ Figures in parentheses are standard errors obtained from the least-squares fit.

**Table 2:** Ab initio equilibrium geometries of H_4_C···ClF, CH_4_, and ClF optimised at the CCSD(T)(F12c)/cc-pVTZ-F12 level of theory.

Molecule	H_4_C···ClF ^a^ A B	CH_4_	ClF
*r*(C–H_1_)/Å	1.0871 1.0872	1.0876	-
*r*(C–H*_n_*)/Å (*n* = 2,3,4)	1.0887 1.0890	1.0876	-
∠Cl···C–H_1_/°	180.00 180.39	-	-
∠H_1_ –C–H*_n_*/° (*n* = 2,3,4)	109.68 109.67	109.47	-
*r*(C···Cl)/Å	3.1914 3.1979	-	-
*r*(Cl–F)/Å	1.6294 1.6308	-	1.6275

^a^ Column A refers to results when ∠Cl···C–H_1_ is set to 180.00°, while the results in column B were obtained when this angle was optimised. Column A is assumed to be the best geometry.

**Table 3:** Distances *r*_cm_ and *r*(C···Cl), oscillation amplitudes $\phi_{\mathrm{av}}{=\cos}^{-1}{\langle \cos^{2}\phi \rangle }^{\frac{1}{2}}$, and intermolecular stretching force constants *k*_σ_ determined for H_4_C···ClF ^a^.

Property	H_4_C···^35^ClF		H_4_C···^37^ClF	
	State *A*	State *F*	State *A*	State *F*
$\phi_{\mathrm{av}}{=\;\cos}^{-1}{\langle \cos^{2}\phi \rangle }^{\frac{1}{2}}$/°	7.1	7.2	7.1	7.2
*r*_cm_/Å	3.8510	3.8704	3.8302	3.8496
*r*(C···Cl)/Å	3.2766	3.2960	3.2763	3.2957
*k_σ_*/(N m^−1^)	3.77(3)	4.54(4)	3.76(2)	4.55(4)

^a^ See Figure 5 for a definition of *r*_cm_, *r*(C···Cl) and angle ϕ. ^b^ The errors in all quantities listed here were generated by the errors in the spectroscopic constants when using Equations (2) and (3) and are insignificant, except for *k_σ_* for which the quoted error is that transmitted from the error in *D_J_*. For all quantities, the errors arising from assumptions in arriving at Equations (2) and (3) are unknown.

**Table 4:** Some properties of CH_4_ and ClF.

Property	CH_4_	^35^ClF	^37^ClF
*B*_0_/MHz	157,122.6142(15) ^a^	15,418.251(5) ^b^	15,125.652(5) ^b^
χ_0_(Cl)/MHz		−145.87182(3) ^c^	−114.96131(6) ^c^
*r*_0_/Å)	1.093987 ^d^	1.63176 ^e^	1.63173 ^e^
*r*/Å	0.000000 ^f^	0.57444 ^g^	0.55393 ^g^

^a^ Ref. [39] ^b^Ref. [40] ^c^ Ref. [38] ^d^ Calculated using $r_{0}={\left\{\frac{3}{8\;}(I_{b}^{{\mathrm{CH}}_{4}}/m_{H})\right\}}^{\frac{1}{2}}$ and the conversion factor $I_{b}^{{\mathrm{CH}}_{4}}B_{0}=505379.005\;\mathrm{MHz}.u{Å}^{2}$. ^e^ Calculated from *B*_0_ using the equation $r_{0}={\left\{\left(I_{b}^{\mathrm{ClF}}/\mu \right)\right\}}^{\frac{1}{2}},$ where $\mu =\;m_{\mathrm{Cl}}m_{F}{\left(m_{\mathrm{Cl}}+\;m_{F}\right)}^{-1}$ and the conversion factor $I_{b}^{\mathrm{ClF}}B_{0}=505379.005\;\mathrm{MHz}.u{Å}^{2}$. ^f^ Distance of C from the centre of mass of CH_4_. ^g^ Distance of Cl from the ClF mass centre for this isotopologue.

## Supplementary Materials

The following are available online. Figure S1: Electrostatic potential of CH_4_ calculated at MP2/aug-cc-pVTZ level; Figure S2: Optimized geometry of the CH_4_…FCl hydrogen bonded geometry, including the bond critical point; Table S1: Rotational transitions for the title complex and their fit; Table S2: AIM parameters for the H_4_C…ClF, H_4_C…HCl and CH_4_…FCl complexes; Table S3: Cartesian coordinates for the optimized geometries of the three complexes; Table S4: Rotational constants for the H_4_C…ClF and CH_4_…FCl complexes; Table S5 and S6: NBO results for H_4_C…ClF and CH_4_…HCl complexes.
