# Supplementary material for: Isolation of a Halogen-Bonded Complex Formed between Methane and Chlorine Monofluoride and Characterisation by Rotational Spectroscopy and Ab Initio Calculations
Source: Molecules. 2019 Nov 22;24(23):4257. doi: 10.3390/molecules24234257 (PMC6930488; doi:10.3390/molecules24234257)
Supplement: Supplementary file 1 [file molecules-24-04257-s001.zip › Supplementary Material/TablesS2_S6_FigureS1_S2.docx]

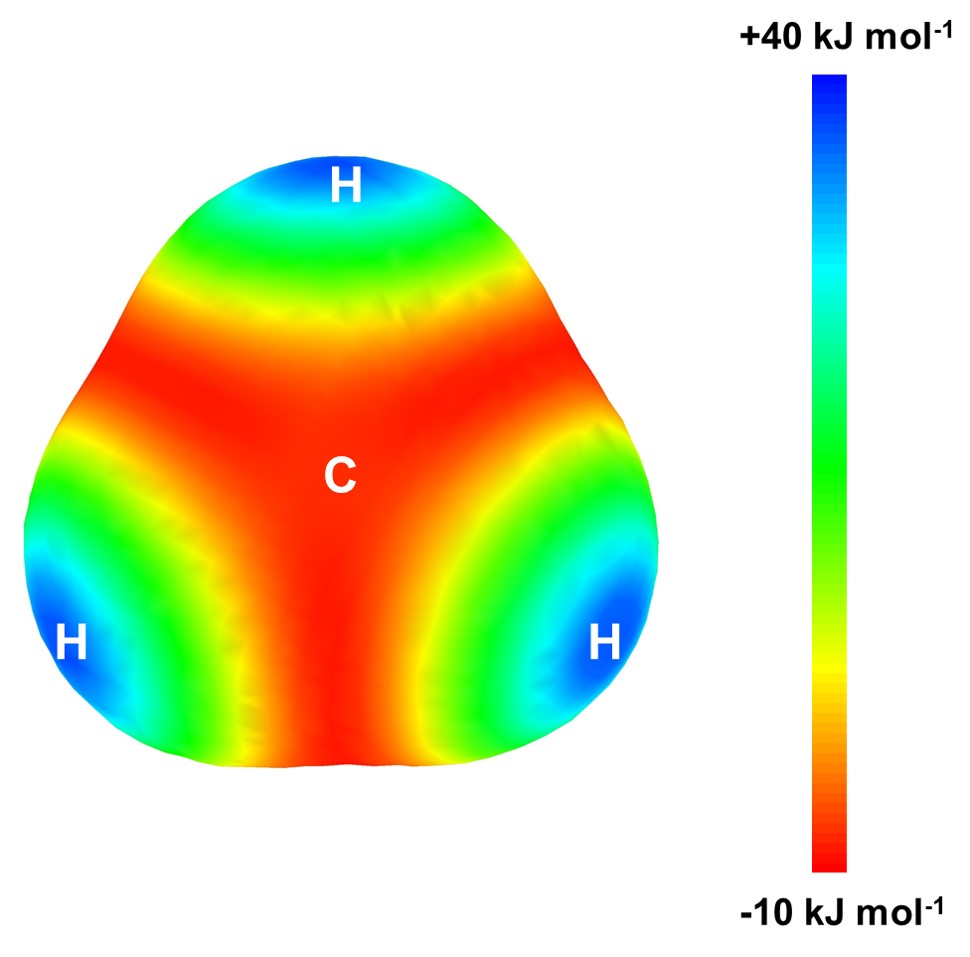


Figure S1: The electrostatic potential of CH_4_ calculated at the MP2/Aug-cc-pVTZ level of calculations. It is very similar to that calculated at MP2/6-311++G** level given in Figure 1 in the main text.


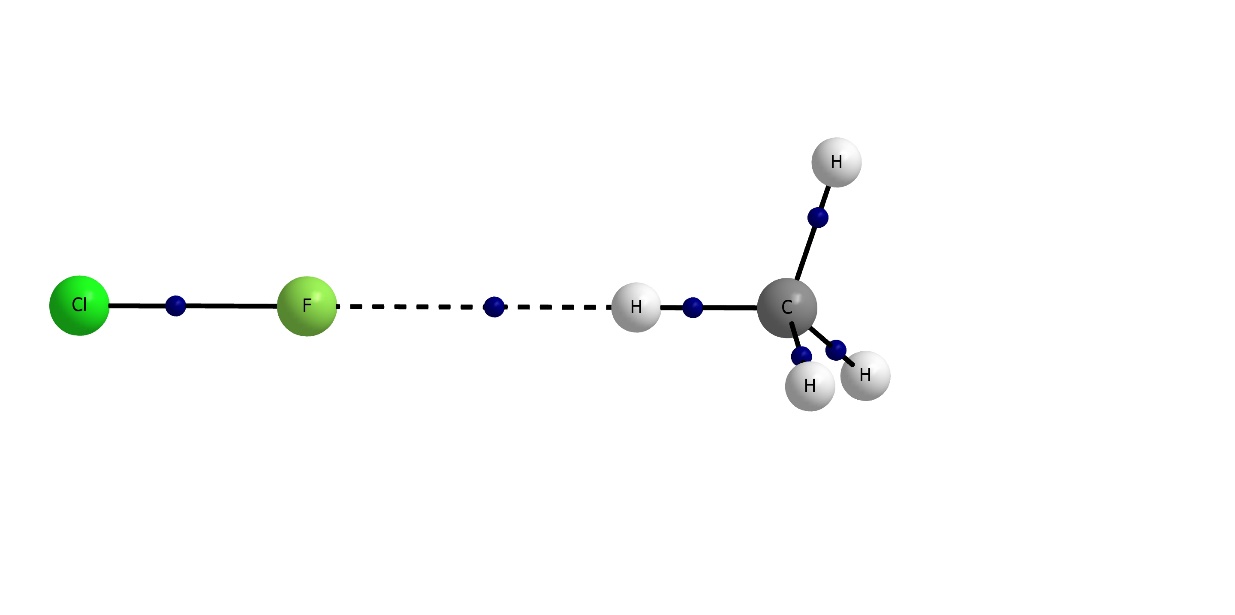


Figure S2. The result of an AIM calculation on Cl-F…H-C hydrogen bonded geometry of methane-ClF complex. The wavefunctions for the geometry optimised at the MP2(Full)/Aug-cc-pVTZ level were used for the AIM calculations.

Table S2. Important parameters obtained from Atoms in molecules analysis of halogen bonded H_4_CClF and hydrogen bonded H_4_CHCl complexes. All the values are for the intermolecular bond critical point (MP2(Full)/Aug-cc-pVTZ level).

|  | At the intermolecular bond critical point | | | | |
| --- | --- | --- | --- | --- | --- |
|  | | | H_4_CClF (H-C...Cl-F bonded) | ClF...H_4_C (Cl-F...H-C bonded) | H_4_CHCl |
| Electron density, ρ in a.u. | | | 0.0085 | 0.0071 | 0.0107 |
| Laplacian of electron density, ∇^2^ρ in a.u. | | | +0.0411 | +0.0349 | +0.0407 |
| Eigen values of Hessian of electron density | | λ_1_ | -0.0023 | -0.0071 | -0.0073 |
|  |  | λ_2_ | -0.0023 | -0.0071 | -0.0073 |
|  |  | λ_3_ | 0.0458 | 0.0491 | 0.0553 |
| \| λ_1_\|/ λ_3_ | | | 0.0502 | 0.1446 | 0.1320 |
| Potential electron energy density (V) | | | -0.0063 | -0.0050 | -0.0074 |
| Kinetic electron energy density (G) | | | 0.0083 | 0.0069 | 0.0088 |
| \|V\|/G | | | 0.7590 | 0.7246 | 0.8409 |

Table S3. Coordinates for the optimised geometries of various complexes at MP2(Full)/Aug-cc-pVTZ level of theory.

| **H_4_C...ClF (H-C...Cl-F bonded)** | | | |
| --- | --- | --- | --- |
|  | x (Å) | y (Å) | z (Å) |
| C | 0.00000 | 0.00000 | -2.62826 |
| H | 0.00000 | 1.02541 | -2.27428 |
| H | -0.88803 | -0.51270 | -2.27428 |
| H | 0.88803 | -0.51270 | -2.27428 |
| H | 0.00000 | 0.00000 | -3.71274 |
| F | 0.00000 | 0.00000 | 2.08234 |
| Cl | 0.00000 | 0.00000 | 0.44495 |
| **H_4_C...ClF (Cl-F...H-C bonded)** | | | |
| C | 3.26320 | 0.00000 | 0.00019 |
| H | 3.62457 | 0.88551 | -0.51103 |
| H | 3.62457 | -0.88570 | -0.51070 |
| H | 2.17994 | 0.00000 | -0.00053 |
| H | 3.62356 | 0.00019 | 1.02303 |
| F | -0.18565 | 0.00000 | -0.00067 |
| Cl | -1.82123 | 0.00000 | 0.00024 |
|  |  |  |  |
| **H_4_C...HCl (H-C...H-Cl bonded)** | | | |
| C | 2.33791 | 0.00004 | 0.00000 |
| H | 1.98492 | 0.51739 | 0.88616 |
| H | 1.98495 | 0.50876 | -0.89115 |
| H | 1.98500 | -1.02610 | 0.00498 |
| H | 3.42210 | 0.00007 | 0.00002 |
| H | -0.04908 | -0.00014 | 0.00000 |
| Cl | -1.32165 | -0.00003 | 0.00000 |

Table S4. Calculated rotational constants for H-C…Cl-F halogen bonded and Cl-F…H-C hydrogen bonded geometries of methane-ClF complex.

|  | methane-ClF (H-C…Cl-F bonded) | methane-ClF (Cl-F…H-C bonded) |
| --- | --- | --- |
| A/MHz | 158971.82 | 159843.68 |
| B/MHz | 2515.38 | 1758.84 |
| C/MHz | 2515.38 | 1758.84 |

| Donor NBO (i) | Acceptor NBO (j) | E(2) kcal/mol |
| --- | --- | --- |
| From Cl-F to methane | | |
| LP ( 1)Cl 7 | BD*( 1) C 1 - H 2 | 0.12 |
| LP ( 1)Cl 7 | BD*( 1) C 1 - H 5 | 0.10 |
| LP ( 2)Cl 7 | BD*( 1) C 1 - H 2 | 0.11 |
| LP ( 2)Cl 7 | BD*( 1) C 1 - H 5 | 0.10 |
| LP ( 3)Cl 7 | BD*( 1) C 1 - H 3 | 0.24 |
| LP ( 3)Cl 7 | BD*( 1) C 1 - H 4 | 0.24 |
| From methane to ClF | | |
| BD ( 1) C 1 - H 2 | BD*( 1) F 6 -Cl 7 | 0.11 |
| BD ( 1) C 1 - H 3 | BD*( 1) F 6 -Cl 7 | 0.73 |
| BD ( 1) C 1 - H 4 | BD*( 1) F 6 -Cl 7 | 0.73 |
| BD ( 1) C 1 - H 5 | BD*( 1) F 6 -Cl 7 | 0.10 |


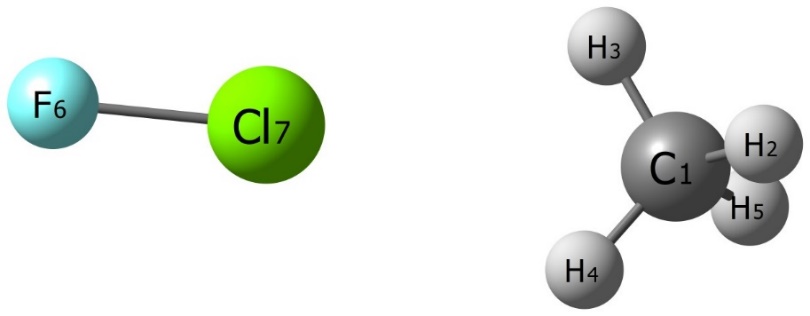
Table S5: Second order perturbation energies calculated due to specific overlaps between the orbitals of CH_4_ and ClF in the complex. The atom numbers are shown in the Figure below.

Table S6: Second order perturbation energies calculated due to specific overlaps between the orbitals of CH_4_ and HCl in the complex.^*^ The atom numbers are shown in the Figure below.

| Donor NBO (i) | Acceptor NBO (j) | E(2) kcal/mol |
| --- | --- | --- |
| From methane to HCl | | |
| BD ( 1) C 1 - H 2 | BD*( 1) H 6 -Cl 7 | 0.70 |
| BD ( 1) C 1 - H 3 | BD*( 1) H 6 -Cl 7 | 0.70 |
| BD ( 1) C 1 - H 4 | BD*( 1) H 6 -Cl 7 | 0.20 |
| BD ( 1) C 1 - H 5 | BD*( 1) H 6 -Cl 7 | 0.19 |


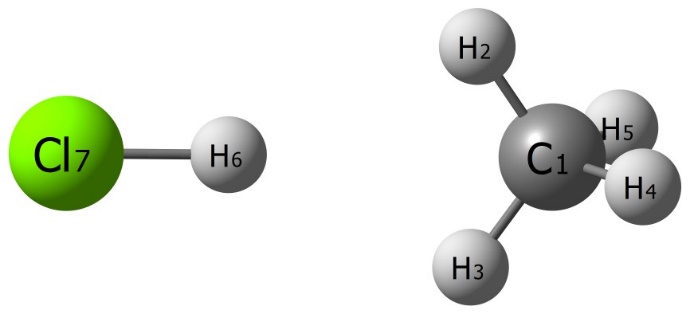
^*^ The E(2) values given for the H_4_C…HCl complex in this table are comparable to that of H_4_C…ClF complex given in Table S5. For the ClF complex, additional stability is provided by the overlap between lone pairs in Cl with those of C-H anti-bonding orbitals. This could contribute to the lower barrier for internal rotation of CH_4_ in the ClF complex compared to that of HCl complex shown in Figure 3 in the main text.
